# The Alternative Complement Pathway Is Activated Without a Corresponding Terminal Pathway Activation in Patients With Heart Failure

**DOI:** 10.3389/fimmu.2021.800978

**Published:** 2021-12-24

**Authors:** Margrethe Flesvig Holt, Annika E. Michelsen, Negar Shahini, Elisabeth Bjørkelund, Christina Holt Bendz, Richard J. Massey, Camilla Schjalm, Bente Halvorsen, Kaspar Broch, Thor Ueland, Lars Gullestad, Per H. Nilsson, Pål Aukrust, Tom Eirik Mollnes, Mieke C. Louwe

**Affiliations:** ^1^ Research Institute of Internal Medicine, Oslo University Hospital, Oslo, Norway; ^2^ Department of Cardiology, Oslo University Hospital, Rikshospitalet, Oslo, Norway; ^3^ Institute of Clinical Medicine, Faculty of Medicine, University of Oslo, Oslo, Norway; ^4^ Department of Immunology, Oslo University Hospital Rikshospitalet and University of Oslo, Oslo, Norway; ^5^ K.G. Jebsen Cardiac Research Center, Center for Heart Failure Research, Faculty of Medicine, University of Oslo, Oslo, Norway; ^6^ Faculty of Health Sciences, K. G. Jebsen Thrombosis Research Center, University of Tromsø – The Arctic University of Norway, Tromsø, Norway; ^7^ Linnaeus Centre for Biomaterials Chemistry, Linnaeus University, Kalmar, Sweden; ^8^ Section of Clinical Immunology and Infectious Diseases, Oslo University Hospital Rikshospitalet, Oslo, Norway; ^9^ Research Laboratory, Nordland Hospital, Bodø, Norway; ^10^ Centre of Molecular Inflammation Research, Norwegian University of Science and Technology, Trondheim, Norway

**Keywords:** heart failure, complement, alternative pathway, complement Factor B, C3bBbP, terminal complement complex

## Abstract

**Objective:**

Dysregulation of the complement system has been described in patients with heart failure (HF). However, data on the alternative pathway are scarce and it is unknown if levels of factor B (FB) and the C3 convertase C3bBbP are elevated in these patients. We hypothesized that plasma levels of FB and C3bBbP would be associated with disease severity and survival in patients with HF.

**Methods:**

We analyzed plasma levels of FB, C3bBbP, and terminal C5b-9 complement complex (TCC) in 343 HF patients and 27 healthy controls.

**Results:**

Compared with controls, patients with HF had elevated levels of circulating FB (1.6-fold, p < 0.001) and C3bBbP (1.3-fold, p < 0.001). In contrast, TCC, reflecting the terminal pathway, was not significantly increased (p = 0.15 vs controls). FB was associated with NT-proBNP, troponin, eGFR, and i.e., C-reactive protein. FB, C3bBbP and TCC were not associated with mortality in HF during a mean follow up of 4.3 years.

**Conclusion:**

Our findings suggest that in patients with HF, the alternative pathway is activated. However, this is not accompanied by activation of the terminal pathway.

## Introduction

Heart failure is a condition in which the heart can’t pump enough blood to meet the body’s needs (1). The development of HF involves several pathological processes, including neurohormonal dysregulation (2) and non-resolving inflammation. Over the last decades, the latter has been shown to be a major player in the pathophysiology of chronic HF (3–5). In addition to the imbalanced cytokine response associated with inflammation, complement dysregulation may play a role in chronic HF (6, 7).

The complement system consists of more than 50 soluble and membrane bound proteins which play a crucial role in innate immunity (8–10). It is activated through the classical, lectin, or alternative pathways, which merge at the central component C3. Complement exerts its functions through several mechanisms: the opsonization of bacteria, the induction of inflammation *via* anaphylatoxins, and the assembly of the terminal complement complex (TCC). TCC, when formed on a cell membrane, can lyse cells. This membrane attack complex is meant to destroy pathogens such as certain Gram-negative bacteria, but can also damage own cells lacking protection (11). In addition to complement’s vital role in the host’s defense against pathogens, complement is important in tissue repair and tissue homeostasis (12). However, whereas complement activation is important for the defense against invading microbes, too strong or too long-lasting activation could harm, rather than protect, the host. This has been shown in various acute and chronic diseases, including cardiovascular diseases (CVD) (13–15).

The alternative pathway of complement activation is continuously activated at a low level and functions as an amplifier of the classical and lectin pathways (16). The low-grade auto-activation, termed “tick over”, constantly generates low levels of C3b that can swiftly activate complement if needed (17, 18). The complement system has been reported to be activated and associated with adverse clinical outcomes in HF (19, 20). We have recently showed that the alternative pathway is dysregulated in patients with chronic HF (21), and that levels of factor B (FB) and Bb are elevated in patients with aortic stenosis (22). However, there is no data on levels of FB and the formation of the C3 convertase in patients with chronic HF. FB is of special interest in HF, because increased cardiac expression of FB has been observed in animal models of cardiac stress (23). Thus, we hypothesized that plasma levels of FB and C3bBbP would be dysregulated in patients with HF compared to healthy controls, and would be associated with disease severity and survival in HF.

## Method

### Study Population

Between 2012 and 2017, we included 343 patients with heart failure referred to the department of cardiology at Oslo University Hospital, Rikshospitalet. Patients with a history of inflammatory or autoimmune disease or cancer; concurrent infections, or recent invasive procedures were not included. Baseline characteristics are shown in **Table 1**. For comparison, blood samples were collected from 27 self-reported healthy subjects of both sexes and similar age (44% female, average age 68 years). Patients were stratified by New York Heart Association (NYHA) functional class at admission, if un-assessed clinical notes were reviewed to correctly classify the patient. Echocardiograms were done as part of the routine clinical evaluation. Availability of parameters of cardiac function by echocardiograms were limited. The etiology of HF was determined based on medical history, echocardiography, and coronary angiography. The etiology was classified as dilated cardiomyopathy (DCM, n = 131), ischemic cardiomyopathy/coronary artery disease (CAD, n = 149), hypertrophic cardiomyopathy (HCM, n=21) and other (n = 42). We obtained mortality data from the Norwegian Cause of Death Registry. Conditions listed in chapter Nine (Codes I.00-I.99) of the International Statistical Classification of Diseases and Related Health Problems 10^th^ edition (ICD-10) were considered as cardiovascular mortality. The study was approved by the Regional Committee for Medical and Health Research Ethics (REK no S-06172) and conducted in accordance with the Declaration of Helsinki. Written informed consent was obtained from all participants.

**Table 1:** Characteristics of the study population.

	Controls (n = 27)	Total population (n = 343)	Survival (n = 184)	Non-survival (n = 159)
**Clinical characteristics**				
Male sex, n (%)	15 (56)	260 (76)*	141 (77)	119 (75)
Age, y	67.5 [66.8 - 69.3]	62.4 [53.0 - 69.8]**	65.1 [56.2 - 70.8]	59.6 [50.9 - 68.4]^#^
BMI, kg/m^2^	24.9 [23.1 - 26.6]	27.4 [23.8 – 30.5]*	27.7 [24.9 - 31.4]	26.6 [23.4 - 29.9]^#^
Etiology: DCM/CAD/HCM/other, n		131/149/21/42	63/88/14/19	68/61/7/23
Time since diagnosis, months		57.5 [8 - 110]	60.0 [9 - 108]	54.5 [8 - 120]
NYHA: I+II/III/IV, n		123/188/28	75/97/10	48/91/18
DM, n (%)		74 (22)	36 (20)	38 (24)
Hypertension, n (%)		98 (30)	57 (31)	41 (26)
Previous MI, n (%)		141(41)	82 (45)	59 (37)
COPD, n (%)		46 (13)	29 (16)	17 (11)
**Echocardiography**				
LVEF, %		25 [20 - 35]	25 [20 - 33]	25 [20 - 35]
**Biochemistry**				
WBC, 10^9^/L	5.2 [4.6-6.4]	7.7 [6.4 - 9.2]***	7.7 [6.2 - 9.2]	7.7 [6.5 - 9.1]
eGFR, mL/min/1.73^2^	86 [75 - 90]	67 [49 - 89]**	67[49 - 89]	66 [48 - 89]
Cholesterol, mmol/L	5.7 [5.3 – 6.6]	4.1 [3.3 – 4.8]***	4.1 [3.3 - 5.0]	4.1 [3.2 – 4.9]
NT-proBNP, pmol/L	9 [4.4 - 14]	230 [102 – 455]***	191[86.75 – 382.5]	278 [133 - 592] ^##^
CRP, mg/L	1.2 [0.7 - 3.2]	3.2 [1.4 – 7.2]**	2.5 [1.1 – 5.7]	4.0 [2.0 - 8.6] ^###^
**Medication, n (%)**				
ACE inhibitors/ARBs		317 (93)	143 (78)	115 (72)
β-Blockers		318 (93)	170 (92)	148 (94)
Diuretics		288 (84)	147 (80)	141 (89)*
Oral Anticoagulants		192 (56)	96 (52)	96 (60)
Antiplatelet therapy		180 (53)	102 (55)	78 (50)
Statins		209 (61)	115 (63)	94 (60)

BMI, body mass index; DCM, dilated cardiomyopathy; CAD, coronary artery disease; HCM, hypertrophic cardiomyopathy; NYHA, New York Heart Association functional class; DM, diabetes mellitus; MI, myocardial infarction; COPD, chronic obstructive pulmonary disease; LVEF, left ventricular ejection fraction; WBC, white blood cell count; eGFR, estimated glomerular filtration rate; NT-proBNP, N-terminal pro–B-type natriuretic peptide; CRP, C-reactive protein; ACE, angiotensin-converting enzyme; ARB, angiotensin receptor blocker. Values are presented as frequency (%) or median [interquartile range] as appropriate. *p < 0.05, **p<0.01, ***p<0.001 patients vs controls, ^#^p < 0.05, ^##^p<0.01, ^###^p<0.001 non-survival vs survival.

### Biochemistry and Blood Sampling

Peripheral venous blood was drawn into sterile EDTA tubes, immersed in melting ice and centrifuged within 20 minutes at 2000*g* for 20 minutes to gain platelet-poor plasma. Plasma was stored in multiple at −80°C. C-reactive protein (CRP) was analyzed using particle enhanced immunobidimetric assay. Troponin T (TnT) (high-sensitivity) and N-terminal pro–B-type natriuretic peptide (NT-proBNP) were analyzed using an electrochemiluminescence immunoassay from Roche Diagnostica (Basel, Switzerland). The estimated glomerular filtration rate (eGFR) was obtained using the CKD-EPI-formula (24).

### Measurements of Circulating Complement Factors in HF Patients

FB levels were quantified by ELISA using plasma diluted 1:400, as described by Shahini et. al (22). The coating monoclonal antibody recognizes the common epitope on both the native FB and the activated Ba fragment, and it does not distinguish between active and inactive fragments. As a result, this assay only reflects the total amount of FB that is present. TCC was measured by ELISA using a monoclonal antibody aE11 reacting with a neoepitope exposed in C9 when incorporated in TCC, in a modified version of the method described by Mollnes et al. (25, 26) C3bBbP was measured as previously described (27), using a monoclonal antibody (human Factor P#2 catalogue no. A235, Quidel, San Diego, CA) as coating antibody and a polyclonal anti-human C3c antibody (catalogue no. OSAP194E0002V, Dade Behring, Deerfield, IL) for the detection of bound C3bBbP. The results for FB, C3bBbP and TCC are given in complement activating units (CAU)/mL related to a standard of complement activated human serum defined to contain 1000 CAU/ml as detailed in Bergseth et al. (26) To assess activity in the classical and the lectin pathways of complement, we measured C4d in plasma (1:100 dilution) from a random selection of patients (n = 72) and controls (n= 14). The C4d ELISA was performed using a kit detecting a unique cleavage epitope on C4d (COMPL C4d RUO, Svar Life Science AB Malmö, Sverige).

### Statistical Analysis

Differences in complement levels between groups were analysed by ANCOVA, adjusting for age, sex and BMI. Due to skewed distribution, the factors were log-transformed prior to analysis. Differences in eGFR and aetiology between groups were analysed with the Mann-Whitney U tests and Pearson’s chi-squared test as appropriate. Associations between variables were assessed by means of Spearman correlation coefficient. To identify predictors of complement levels we performed univariate regression analysis and variables that were associated with complement levels (p<0.05) were included in a stepwise multivariable regression analysis. Kaplan-Meier analysis with log rank test was used to analyse all-cause/anticipated mortality stratified by quartiles of FB and C3bBbP. P-values are 2-sided and considered significant when <0.05.

## Results

### Circulating Levels of FB and C3bBbP Are Increased in Patients With Heart Failure

**Figure 1:**
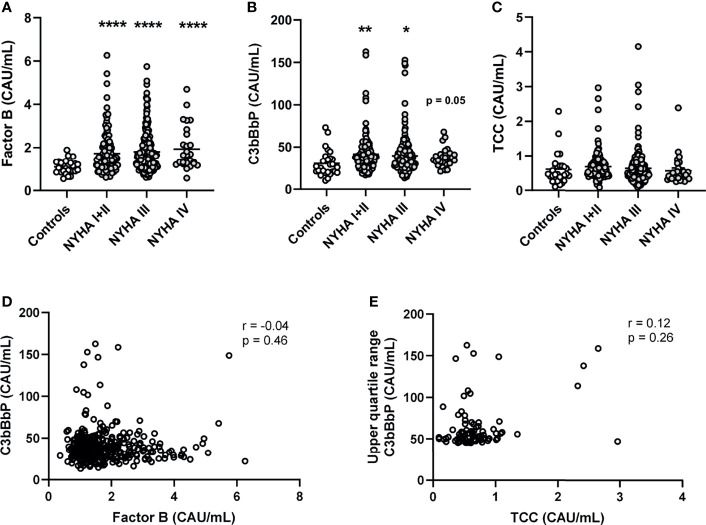
Plasma levels of alternative complement pathway components Factor B and C3bBbP are increased in patients with heart failure and do not correlate. Plasma levels of **(A)** complement factor B (FB), **(B)** C3bBbP, and **(C)** TCC were measured in 343 heart failure patients and 27 controls. Correlation between plasma levels of **(D)** Factor B and C3bBbP, and **(E)** C3bBbP and TCC levels. NYHA, New York Heart Association functional class; TCC, terminal complement complex; Values are mean ± SEM *p < 0.05, **p < 0.01, ****p < 0.0001.

Compared with healthy controls, patients had significantly increased levels of FB (1.6-fold for all NYHA classes combined, **Figure 1A**). C3bBbP levels were 1.3-fold increased in the total HF population (**Figure 1B**). In contrast, TCC levels were not significantly increased in patients compared with controls (**Figure 1C**), suggesting that the terminal pathway was less activated than the alternative pathway.

### Circulating Levels of C3bBbP Do Not Correlate With FB or TCC Levels

In patients with HF, there was no significant correlation between the levels of FB and C3bBbP (**Figure 1D**), indicating that the level of FB increased independently from the formation of C3 convertase. Also, even when comparing the highest quartile of C3bBbP levels in patients with HF (e.g. the patients with low or normal C3bBbP levels are excluded) with their respective TCC levels, there was no significant correlation (**Figure 1E**). When we look more in depth into the different HF aetiologies, patients with DCM (n = 131) and CAD (n = 149) had elevated levels of FB and C3bBbP. The levels of FB were particularly high in patients with CAD (**Supplemental Figure S1**). Lastly, levels of C4d, a complement activation marker for the classical and lectin- pathways, were not significantly increased in HF patients compared with healthy controls (**Supplemental Figure S2**), suggesting that the classical and lectin pathway are not activated in these patients.

### Circulating Levels of FB Are Associated With Measures of Cardiac and Kidney Function

**Figure 2:**
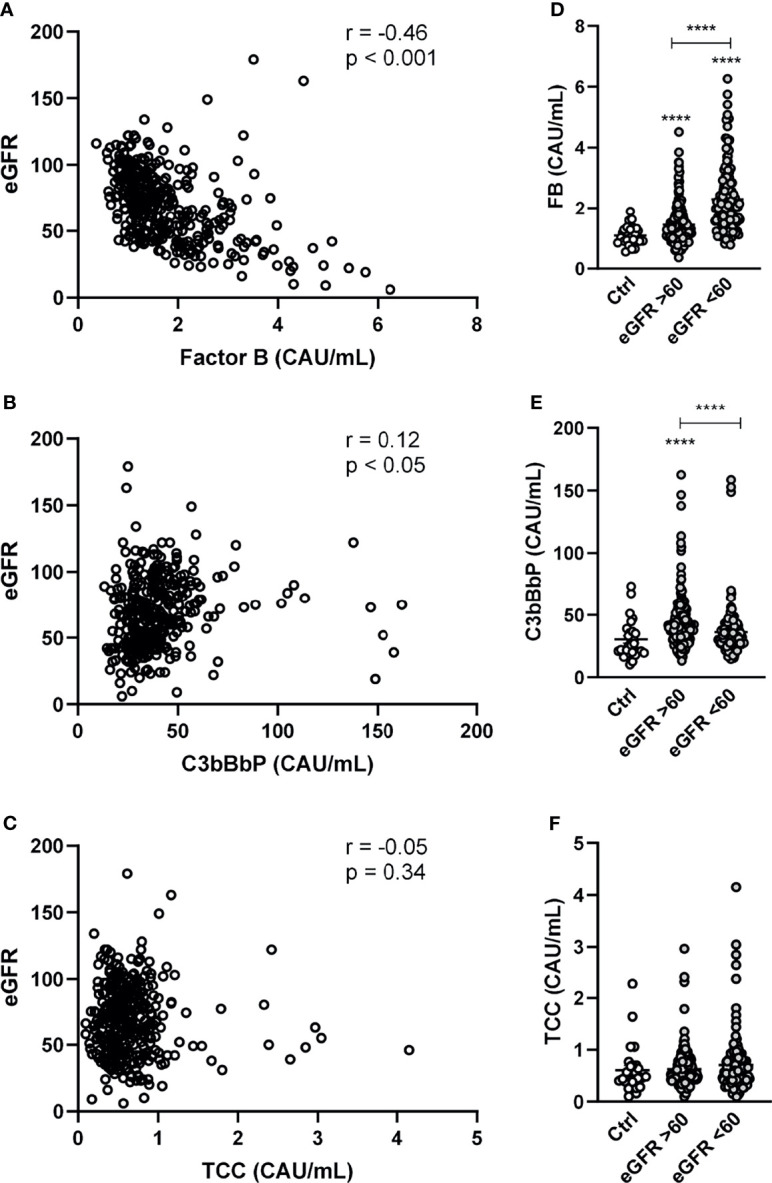
Factor B and C3bBbP are partly associated with impaired kidney function. Correlation between estimated glomerular filtration rate (eGRF) and plasma levels of **(A)** factor B (FB) **(B)** C3bBbP and **(C)** TCC. Levels of **(D)** FB, **(E)** C3bBbP and **(F)** TCC in healthy controls and in HF patients with normal (i.e. eGFR > 60) or deteriorated (i.e. eGFR < 60) kidney function. Values are mean ± SEM ****p < 0.0001.

The relationship between complement components and clinical and biochemical markers of cardiac function are summarized in **Supplemental Table S1**. NT-proBNP correlated positively with FB and negatively with C3bBbP. FB, but not C3bBbP, was positively correlated with TnT and CRP. eGFR was negatively correlated with FB and positively with C3bBbP, but the latter correlation was weak (**Figures 2A, B**). However, there was no correlation between eGFR and TCC (**Figure 2C**). When dividing patients with HF in to normal (i.e. eGFR > 60) or deteriorated (i.e. eGFR < 60) kidney function a step-wise increase in FB levels in relation to kidney dysfunction was observed (**Figure 2D**). Levels of C3bBbP were only increased in patients with preserved kidney function (**Figure 2E**), whereas TCC levels did not differ (**Figure 2F**).

### Predictors of FB, C3bBbP and TCC in HF: Multivariate Analyses

To analyze the determinants of the different complement factors, we next performed univariate and stepwise multivariable regression to find the factors associated with complement levels (**Table 2**). Multiple demographic and biochemical indices of importance for HF were associated with FB with low eGFR and cholesterol and high CRP and NT-proBNP remaining significant in stepwise regression. For C3bBbP, male sex, higher eGFR and lower NT-proBNP were associated with higher levels. TCC was correlated positively with CRP and negatively with NYHA class.

**Table 2:** Univariate and Multivariate analysis of FB, C3bBbP and TCC.

	FB		C3bBbP		TCC	
	Uni	Multi	Uni	Multi	Uni	Multi
Male sex	0.07		0.19**	0.19**	0.02	
Age	0.24**	0.13*	-0.10		0.04	
BMI	-0.03		0.13*		0.01	
Aetiology CAD	0.15**		0.00		0.03	
Duration HF	0.17**		0.04		-0.03	
NYHA	0.07		-0.05		-0.13*	-0.13*
DM	0.07		-0.08		-0.02	
Hypertension	0.09		-0.08		0.02	
Previous MI	0.11*		-0.01		-0.03	
COPD	0.01		-0.02		-0.02	
LVEF	0.02		-0.06		0.03	
WBC	-0.01		0.06		0.00	
eGFR	-0.52**	-0.38**	0.17**	0.14**	-0.04	
Cholesterol	-0.30**	-0.20**	0.03		0.01	
NT-proBNP	0.37**	0.17**	-0.18**	-0.14*	-0.04	
CRP	0.25**	0.12*	0.00		0.12*	0.13*

Uni, univariate analysis; Multi, multivariate analysis; BMI, body mass index; CAD, coronary artery disease; HF, heart failure; NYHA, New York Heart Association functional class; NYHA, New York Heart Association functional class; MI, myocardial infarction; COPD, chronic obstructive pulmonary disease; LVEF, left ventricular ejection fraction; WBC, white blood cell; eGFR, estimated glomerular filtration rate; NT-proBNP, N-terminal pro–B-type natriuretic peptide; CRP, C-reactive protein. For multivariable analysis standardized beta is shown. *p<0.05, **p<0.005.

### FB, C3bBbP and TCC Levels Do Not Predict Long Term Mortality in HF Patients

**Figure 3:**
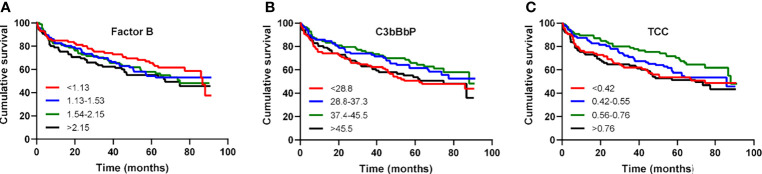
Factor B, C3bBbP and TCC levels are not associated with all-cause mortality in patients with heart failure. Kaplan–Meier survival analysis of cumulative survival in relation to quartile levels of **(A)** factor B, **(B)** C3bBbP and **(C)** TCC.

Over a mean follow-up of 4.3 years, 61 patients underwent heart transplantation and 98 patients died, of which 72% died due to diseases of the circulatory system; ischemic heart disease and acute myocardial infarction being the most frequent of them. Levels of FB, C3bBbP or TCC were not associated with the combined outcome of heart transplant or death (**Figures 3A–C**).

## Discussion

In the present study we show a substantial activation of the alternative complement pathway as reflected by an increase in the C3 convertase C3bBbP in patients with HF. Moreover, we show that FB, a crucial component of the alternative pathway, is upregulated in HF patients and particularly in patients with ischemic cardiomyopathy. However, these changes were not reflected in survival differences of HF patients when focussing on levels of FB, C3bBbP or TCC.

Recently we showed that the alternative pathway is dysregulated in HF as reflected by increased levels of factor D and properdin and decreased levels of factor H (21). However, data on FB in CVD is scarce. Yasuda et al. observed no increase of native FB in a study with a small number of patients with coronary stenosis or angina (28). However, patients with acute myocardial infarction had markedly increased values of the activation fragment Bb indicating alternative pathway activation (28). This is in contrast to a study by Hertle et al. which found that Bb was not associated with CVD (29). Lastly, we have previously described increased levels of FB and Bb in patients with aortic stenosis (22), a common valvular disease and a possible underlying cause of HF. However, none of these studies were focusing purely on patient with HF. To the best of our knowledge, our study is the first to show that FB is elevated in HF and particularly in patients with ischemic heart disease. FB was associated with NT-proBNP and TnT, implying a relation to myocardial damage. However, FB was not associated with adverse clinical outcomes during follow-up.

Patients with HF had higher levels of C3bBbP than healthy controls. Despite having a relatively low number of controls, the values in these apparently healthy individuals were close to the reference range of C3bBbP, as given by Bergseth et al. (26) The increase in C3bBbP levels suggests an overall increased activity of the alternative pathway in HF patients. FB, however, did not correlate with C3bBbP, suggesting that the increased levels of FB could reflect an ongoing acute phase reaction, consistent with the significant correlation between FB and CRP. The positive correlation between C3bBbP levels and kidney function may seem surprisingly. However, the correlation was weak and should be interpreted with caution. Moreover, multivariate analyses show that in addition to eGFR, low NT-proBNP and male gender were the strongest predictors of C3bBbP with gender as the strongest factor.

Somewhat surprisingly, the HF patients in this cohort on average had normal TCC levels compared to controls, and, thus there was no evidence of increased terminal pathway activation. This is contrary to our previous findings in other HF cohorts, where we reported increased levels of TCC (6, 21). At several points the previous and current two, not overlapping, populations differ; among others, our population is older and has a higher frequency of comorbidities such as diabetes and hypertension. The discrepancy between these studies could potentially reflect that the previous HF cohort comprised of more end-stage HF (e.g., patients with NYHA class IV, 8% in the current cohort vs. 27% in the previous cohort). Nonetheless, why TCC levels are normal in the presence of enhanced activation of the alternative pathway, is not clear. However, in some patients with nephritic factors, early-phase activation may not lead to activation of the terminal pathway (30).

Whereas complement activation in the circulation is clearly of interest, data on activation of the alternative complement pathway within the myocardium in HF patients are essential lacking. There a few clinical studies on complement disposition within the myocardium and all of them on myocardial infarcted tissue. The alternative pathway is only briefly brought up by Väkevä et al. (31), describing that properdin was not observed in the infarcted lesions using immune-fluorescence microscopy. However, it is also stated that the absence of properdin does not completely rule out the involvement of the alternative pathway, since deposition can be hard to judge by immunofluorescence microscopy. In addition, and contrary to observations in plasma samples from the population of our HF patients, they observed in cardiac tissue the presence of Clq, C4, and C5, C6, C8, C9 including the membrane attack complex (C5b-9/TCC) in complement deposits, reflecting the classical and terminal pathways, respectively. The activation of these pathways in myocardial infarcted tissue is also confirmed by Yasojima et al. with deposits of C4d, C3d, and the membrane attack complex (C5b-9) on damaged cardiac myocytes (32). In both publications the studied patients died from an acute myocardial infarction, which may not reflect the situation in patients with chronic HF as in the present study.

There are some limitations that should be considered when interpreting the current study. The number of controls as well as patients with severe HF was limited. Furthermore, although statistically significant, some of the correlation coefficients were rather low. Moreover, we lack data on complement deposition with the failing myocardium.

In summary, our results show that circulating levels of FB and C3bBbP are elevated in patients with HF suggesting a role for activation of the alternative complement pathway in HF. However, the pathophysiological consequences of these findings are unknown, since complement factors did not correlate with disease severity.

## Data Availability Statement

The Regional Ethics Committee in Norway (REK Sør-Øst) approved the conduction of the study. A condition for approval was that privacy concerns were respected and that data were not made publicly available. However, excerpts of de-identified data relevant to the study can be made available upon reasonable request. Requests to access the datasets should be directed to m.c.louwe@ous-research.no.

## Ethics Statement

The studies involving human participants were reviewed and approved by Regional Committee for Medical and Health Research Ethics. The patients/participants provided their written informed consent to participate in this study.

## Author Contributions

PA, LG, TM, and ML conceived and designed the research. EB, CB, RM, KB, and LG established the biobank, included patients and collected the material. MH organized the database. CS, BH, and TM contributed to reagents and material. AM, NS, and CS prepared the samples and performed experiments. MH, TU, and ML performed statistical analysis. TU, PN, PA, TM, and ML interpreted the results. MH, PA, TM, and ML wrote the first draft of the manuscript. All authors contributed to manuscript revision, read, and approved the submitted version.

## Funding

This work was supported by the South-Eastern Norway Regional Health Authority (grant 2019058 to ML).

## Conflict of Interest

NS is currently an employee of Johnson and Johnson; the current work was conducted when she was employed by Oslo University Hospital.

The remaining authors declare that the research was conducted in the absence of any commercial or financial relationships that could be construed as a potential conflict of interest.

## Publisher’s Note

All claims expressed in this article are solely those of the authors and do not necessarily represent those of their affiliated organizations, or those of the publisher, the editors and the reviewers. Any product that may be evaluated in this article, or claim that may be made by its manufacturer, is not guaranteed or endorsed by the publisher.

## Abbreviations

CAD, coronary artery disease; CRP, C-reactive protein; CVD, cardiovascular disease; DCM, dilated cardiomyopathy; eGFR, estimated glomerular filtration rate; HF, heart failure; NT-proBNP, N-terminal pro–B-type natriuretic peptide; NYHA-class, New York Heart Association class; TCC, terminal complement complex; TnT, troponin-T.
