# Supplementary figures and images for: The Alternative Complement Pathway Is Activated Without a Corresponding Terminal Pathway Activation in Patients With Heart Failure
Source: Front Immunol. 2021 Dec 24;12:800978. doi: 10.3389/fimmu.2021.800978 (PMC8738166; doi:10.3389/fimmu.2021.800978)

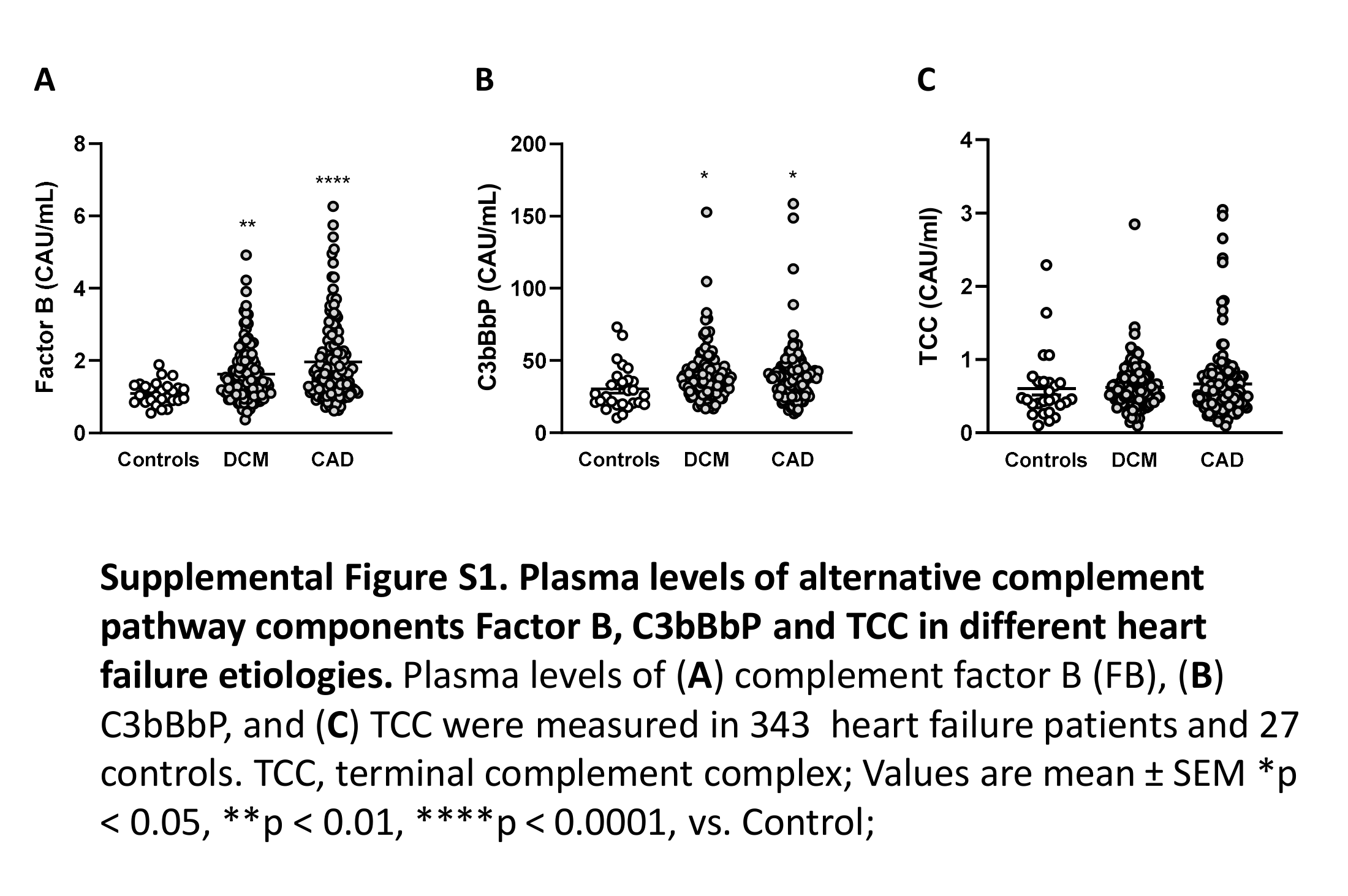

Supplement: Supplementary file 1 [file Image_1.tiff]

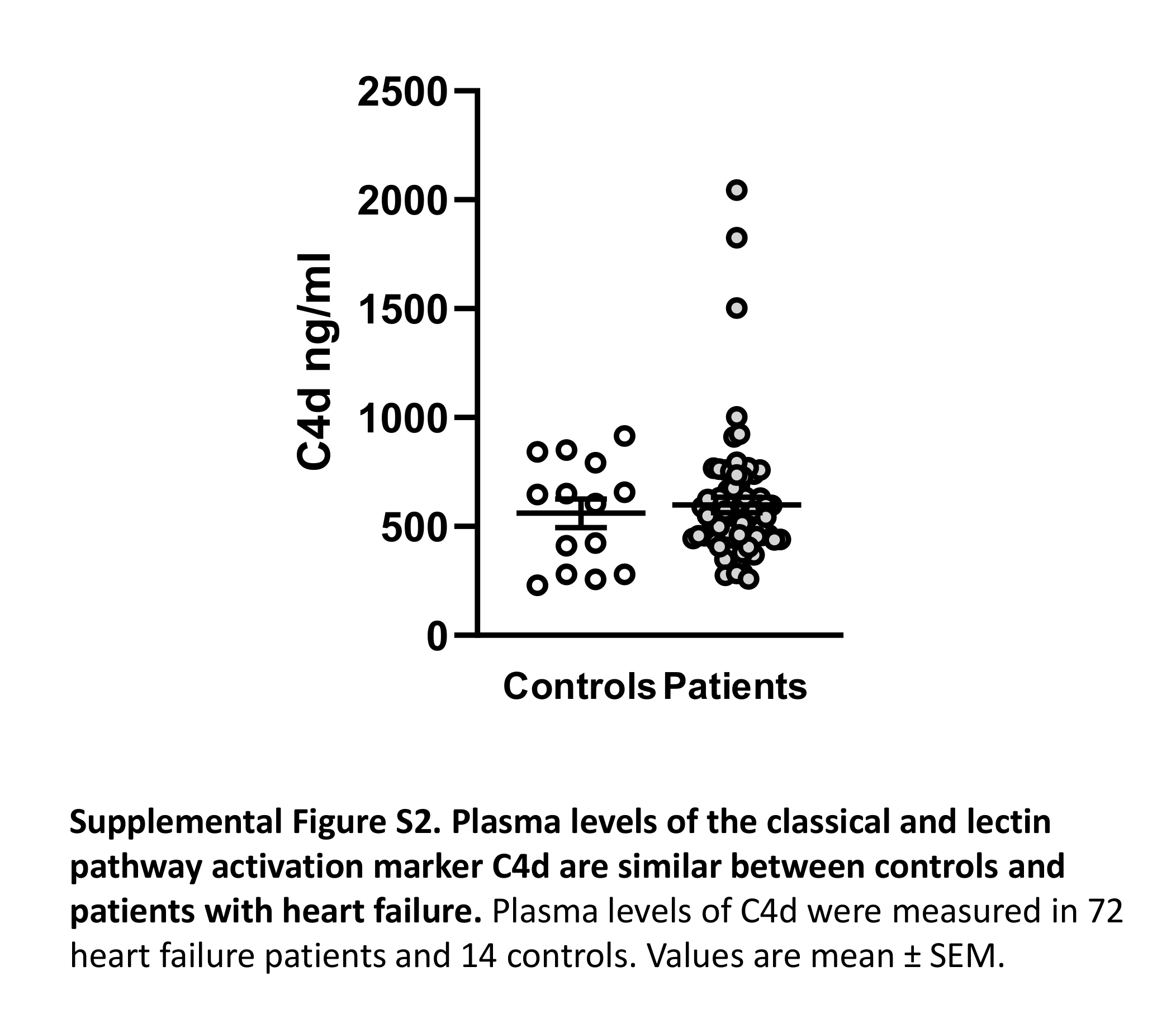

Supplement: Supplementary file 2 [file Image_2.tiff]
