# Supplementary material for: The Alternative Complement Pathway Is Activated Without a Corresponding Terminal Pathway Activation in Patients With Heart Failure
Source: Front Immunol. 2021 Dec 24;12:800978. doi: 10.3389/fimmu.2021.800978 (PMC8738166; doi:10.3389/fimmu.2021.800978)
Supplement: Supplementary file 3 [file Table_1.docx]

**Supplemental table S1. Associations of FB and C3bBbP with heart function and biochemical parameters.**

|  | | **FB** | | | **C3bBbP** | |
| --- | --- | --- | --- | --- | --- | --- |
|  | ***r*** | | ***p value*** | ***r*** | | ***p value*** |
| NT-proBNP | 0.38 | | **< 0.001** | -0.18 | | **< 0.001** |
| LVEF | -0.06 | | 0.31 | -0.06 | | 0.28 |
| TnT | 0.38 | | **< 0.001** | -0.10 | | 0.07 |
| CRP | 0.39 | | **< 0.001** | -0.03 | | 0.58 |
| eGFR | -0.46 | | **< 0.001** | 0.12 | | **< 0.05** |

Abbreviations: FB, complement factor B; NT-ProBNP, N-Terminal pro-B-type natriuretic peptide; LVEF, left ventricular ejection fraction; TnT, troponin T; CRP, C-reactive protein; eGFR, estimated glomerular filtration rate. p-values < 0.05 are in bold.
